# Supplementary material for: Management and Outcome of Young Women (≤40 Years) with Breast Cancer in Switzerland
Source: Cancers (Basel). 2022 Mar 4;14(5):1328. doi: 10.3390/cancers14051328 (PMC8909610; doi:10.3390/cancers14051328)
Supplement: Supplementary file 1 [file cancers-14-01328-s001.zip › cancers-1583503-supplementary.pdf]

cancers

MDPI

Management and Outcome of Young Women (≤ 40 years) with Breast Cancer in Switzerland

Giacomo Montagna, Robin Schaffar, Andrea Bordoni, Alessandra Spitale, Daniela Andreina Terribile, Lorenzo Rossi, Yvan Bergeron, Bernadette W.A. van der Linden, Isabelle Konzelmann, Sabine Rohrmann, Katharina Staehelin, Manuela Maspoli-Conconi, Jean-Luc Bulliard, Francesco Meani, Olivia Pagani and Elisabetta Rapiti

Table S1. Number of cases per canton per year .

	Number of cases/canton/year									
Year of diagnosis	Zurich	Fribourg	Basel Stadt	Basel Landschaft	Ticino	Vaud	Wallis	Neuchatel	Geneva	Jura
2000	41		14	10	10	33	12	7	16	
2001	52		4	9	12	24	14	5	20	
2002	38		6	7	16	28	12	7	17	
2003	52		9	3	13	35	10	6	22	
2004	62		3	12	22	23	8	8	29	
2005	51		7	10	11	32	8	8	23	3
2006	44	14	9	14	15	32	10	8	22	0
2007	57	11	5	6	9	35	9	6	29	2
2008	70	8	6	5	15	38	10	3	25	1
2009	61	19	7	12	13	38	13	7	20	3
2010	58	21	3	12	17	41	13	6	21	1
2011	39	18	5	10	12	46	9	10	27	1
2012	58	17			14	34	12	5	18	3
2013	65	13			19	32	15	7	28	2
2014	72	10			10	41	12	3	19	1

Table S2. Univariate and multivariable associations between clinicopathological factors and net survival.

	Univariable		Multivariable*	
	Hazard ratio (95% CI)	P	Hazard ratio (95% CI)	P
**Linguistic/geographic/ region**				
German region	Ref			
Latin region	0.73 (0.58-0.90)	**0.003**	0.85 (0.68-1.03)	0.138
**Age group**				
≤ 35	Ref			
>35	0.76 (0.61-0.95)	**0.016**	0.85 (0.67-1.06)	0.153
**Quality of care score**				
1st tertile	Ref			
2nd tertile	0.83 (0.60-1.16)	0.284		
3rd tertile	0.94 (0.69-1.27)	0.670		
100%	0.82 (0.63-1.08)	0.167		
**Period of diagnosis**				
2000-2004	Ref		Ref	
2005-2009	0.66 (0.52-0.85)	**0.001**	0.57 (0.44-0.74)	**< 0.0001**
2010-2014	0.65 (0.48-0.89)	**0.008**	0.58 (0.41-0.80)	**0.001**
**Tumor differentiation**				
Well	Ref		Ref	
Moderately	3.83 (1.61-9.11)	**0.002**	2.84 (1.19-6.75)	**0.018**
Poorly	6.63 (2.82-15.64)	**< 0.0001**	3.92 (1.63-9.41)	**0.002**
Missing	6.00 (2.24-16.10)	**< 0.0001**	3.50 (1.26-9.46)	**0.016**

**Tumor Stage**				
I	Ref		Ref	
II	2.96 (2.05-4.28)	**< 0.0001**	2.60 (1.79-3.74)	**< 0.0001**
III	7.01 (4.77-10.30)	**< 0.0001**	6.13 (4.16-9.05)	**< 0.0001**
Missing	5.44 (2.24-16.10)	**< 0.0001**	4.76 (2.96-7.68)	**< 0.0001**
**Tumor subtype**				
Triple negative	Ref			
Luminal A like	0.38 (0.25-0.56)	**< 0.0001**	0.60 (0.38-0.92)	**0.021**
Luminal B like	0.76 (0.57-1.01)	0.057	0.82 (0.61-1.11)	0.186
HER2+	1.12 (0.72-1.75)	0.602	0.94 (0.61-1.47)	0.780
Unknown	0.66 (0.47-0.94)	**0.02**	0.66 (0.44-0.96)	**0.032**

* adjusted for linguistic/geographic region, age, period of diagnosis, tumor differentiation, stage and subtype.

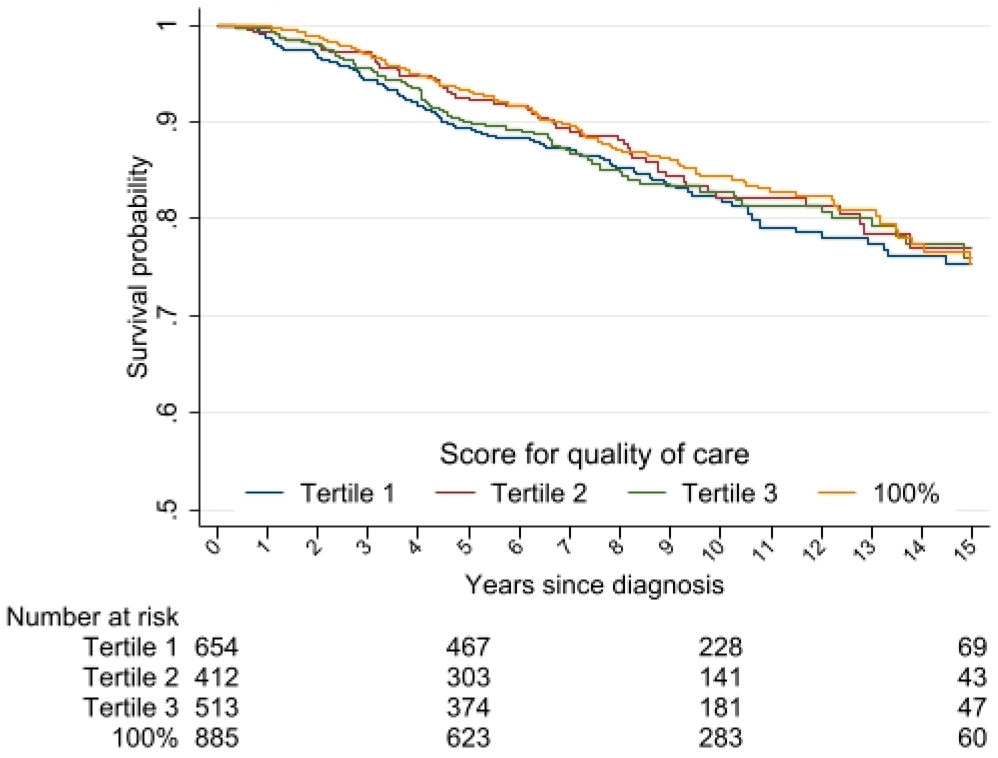

Figure S1. Kaplan-Meier estimates for overall survival by quality-of-care score tertiles.
